# Identification of Candidate Iron Transporters From the ZIP/ZnT Gene Families in the Mosquito *Aedes aegypti*

**DOI:** 10.3389/fphys.2018.00380

**Published:** 2018-04-12

**Authors:** Hitoshi Tsujimoto, Michelle A. E. Anderson, Kevin M. Myles, Zach N. Adelman

**Affiliations:** ^1^Department of Entomology and Agrilife Research, Texas A&M University, College Station, TX, United States; ^2^Department of Entomology and Fralin Life Science Institute, Virginia Tech, Blacksburg, VA, United States

**Keywords:** Aedes, iron transporter, ZIP, ZnT, ferritin

## Abstract

Mosquito-transmitted viral pathogens, such as dengue and Zika, afflict tens of thousands of people every year. These viruses are transmitted during the blood-feeding process that is required for mosquito reproduction, the most important vector being *Aedes aegypti*. While vertebrate blood is rich in protein, its high iron content is potentially toxic to mosquitoes. Although iron transport and sequestration are essential in the reproduction of vector mosquitoes, we discovered that culicine mosquitoes lack homologs of the common iron transporter NRAMP. Using a novel cell-based screen, we identified two ZIP and one ZnT genes as candidate iron transporters in the mosquito *A. aegypti*, the vector of dengue, Zika, and chikungunya. We determined the organ-specific expression pattern of these genes at critical time points in early reproduction. The result indicates modulation of these genes upon blood feeding, especially a ZIP13 homolog that is highly up-regulated after blood feeding, suggesting its importance in iron mobilization during blood digestion and reproduction. Gene silencing resulted in differential iron accumulation in the midgut and ovaries. This study sets a foundation for further investigation of iron transport and control strategies of this viral vector.

## Introduction

In recent years, diseases caused by mosquito-transmitted flaviviruses such as Zika and dengue fever have raised concern internationally (Bhatt et al., 2013; Gatherer and Kohl, 2015; Hennessey et al., 2016). Globally, the incidence of these diseases, especially dengue, show a steady increase over the past 50 years and additional effective measures to reduce the burden of infection are urgently needed (WHO, 2009; Bhatt et al., 2013). *Aedes aegypti* is the major vector of these viruses as well as other viruses and filarial parasites due to its adaptation to domestic settings (Powell and Tabachnick, 2013). Blood feeding is required for *A. aegypti* to initiate egg development and provide nutrients to its offspring; and it is during this act of bloodfeeding that the viruses are transmitted.

Vertebrate blood, while rich in protein, is potentially toxic in large quantities because of its high iron content that can catalyze the formation of oxygen radicals. Mosquitoes have evolved to use vertebrate blood for their reproduction by overcoming this dilemma. Conversely, mosquitoes could use high oxidative stress due to iron in blood meal to control pathogen infection. For example, activation of the Toll immune pathway in *A. aegypti* reduced dengue virus titer which is correlated to reduction of oxidative response genes (Xi et al., 2008); and nitric oxide metabolites by oxyhemoglobin from blood limits development of malaria parasites in the mosquito midgut (Peterson et al., 2007). While digestion of blood proteins is extensively studied, utilization of blood-derived iron in mosquitoes is understudied. In fact, despite iron being an essential nutrient and co-factor for a number of biological processes, metabolism of iron is not fully understood, particularly in insects (Tang and Zhou, 2013). Iron is transported between and within cells in different forms—free ions or bound to protein carriers such as ferritin and transferrin (Pham and Winzerling, 2010; Garrick, 2011). In *Drosophila melanogaster* ionic iron is absorbed through Malvolio (Mvl), a homolog of mammalian NRAMP (natural resistance associated macrophage protein), which is a DMT (divalent metal ion transporter) (Orgad et al., 1998). NRAMP homologs function as iron transporters from bacteria to humans and are highly conserved (Nevo and Nelson, 2006). Despite the potential importance of NRAMP, the only other insect NRAMP to be functionally characterized was that from the malaria vector *Anopheles albimanus* (Martinez-Barnetche et al., 2007). Thus, more detailed molecular mechanisms for iron transport remain to be determined.

NRAMP has also been identified as a gene conferring resistance to intracellular pathogen infection in mammals (Vidal et al., 1993). Recently, Rose et al. (2011) reported that *Drosophila* Mvl serves as a receptor for Sindbis virus (SINV) entry *in vitro* and *in vivo*. The same study showed that suppressing Mvl expression by supplementation of iron reduced SINV infection in *A. aegypti* Aag2 cells as well as *Drosophila* DL1 cells. From this result they concluded that NRAMP is involved in SINV entry into the natural mosquito host. However, despite the availability of several culicine genomes, we were unable to identify an NRAMP ortholog in *A. aegypti* or other culicine mosquitoes. This raised the even larger question of how these bloodfeeding mosquitoes acquire iron from their diet. As a result, we screened members of the ZIP (Zinc-regulated transporter/Iron-regulated transporter-like) and ZnT (Zinc transporters) families for involvement in iron transport in *A. aegypti* under the hypothesis that culicine mosquitoes co-adapted these genes as a functional replacement for NRAMP. We identified three candidate iron transporter genes via an *in vitro* cell-based iron-specific reporter system. To further verify their iron transporting functions, expression analysis and RNAi phenotype assays suggest that these genes indeed related to iron transport in *A. aegypti*.

## Materials and methods

### Sindbis-EGFP replication assay

To generate recombinant Sindbis virus expressing EGFP, the EGFP ORF was cloned into the Sindbis virus expression plasmid pTE/3′2Jmcs as previously described (Myles et al., 2008). Following plasmid linearization, SP6-based RNA transcription and transfection into BHK-21 cells, virus was harvested as described (Myles et al., 2008). For challenge experiments, Aag2 cells were seeded into sealed 25 cm^2^ flasks and incubated with 50 μM of the indicated source of iron for 72 h prior to virus challenge. Cells were infected by gently rocking for 30 min at an MOI of ~0.01, at which time virus suspension was removed and growth media replaced; cells were imaged on a Zeiss Axiovert 200 at 24 and 48 h after infection.

### Plasmid assembly

To develop an iron inducible reporter plasmid, the putative *A. aegypti* ferritin light-chain (AAEL007383) promoter region was amplified using the primers listed in Supplementary Table 1. The resulting amplicon was digested with HindIII/NcoI and ligated into the HindIII/NcoI sites of pGL3-basic (Promega; Madison, WI), yielding the final reporter construct pLCH-FFluc. Construction of the normalization control plasmid pSLfa-PUb-Renilla luciferase was described previously (Haac et al., 2015).

### dsRNA preparation

To generate dsRNA targeting each candidate ZIP and Znt gene, PCR amplicons were generated using Phusion polymerase (New England Biolabs; Ipswich, MA), primers listed in Supplementary Table 1 and cDNA generated from *A. aegypti* Liverpool strain mosquitoes as template. Amplicons were used as a template for either the Replicator RNAi kit (Thermo-Fisher Scientific; Waltham, MA) with T7 polymerase and Phi6 replicase or the MEGASCRIPT T7 RNAi kit (Life Technologies; Carlsbad, CA), with two T7 promoters. After synthesis dsRNAs were nuclease treated to eliminate ssRNA as well as the DNA template, and then purified using the MEGAClear kit (Life Technologies; Carlsbad, CA).

### Cells, transfections, and luciferase assays

For all cell culture experiments, *A. aegypti* Aag2 cells were maintained in Leibovitz's L-15 media (Gibco), supplemented with 10% FBS (Atlanta Biologicals), 2% Tryptose phosphate broth (Gibco), and 1% Pen-strep (Corning) at 28°C.

For the RNAi screen of ZIP and ZnT genes for a role in iron transport, dsRNA transfections were performed using Lipofectamine 2000 (Life Technologies; Carlsbad, CA). Briefly, for each dsRNA, 4 μL Lipofectamine 2000 was diluted into 100 μL Opti-mem I Reduced serum media (Gibco); 1.6 μg dsRNA was diluted into a second 100 μL Opti-mem I. These mixtures were combined in the well of a 12-well plate and incubated for 5 min. Cells were counted, diluted to 5 × 10^6^ cells/ml with 1 mL of cell suspension seeded into each well containing the transfection mix. After 3 days cells were aspirated into fresh media, counted and again diluted to 5 × 10^6^ cells/ml. To introduce the reporter construct and normalization control, transfection mixes were prepared in a 96 well plate as follows: per well 0.5 μL Lipofectamine 2000 in 25 μL Opti-mem I, 100 ng pLCH-FFluc and 100 ng pSLfa/PUb-Renilla luciferase (Haac et al., 2015) in 25 μL Opti-mem I. The DNA/Lipofectamine mix was incubated for 5 min and then 100 μL of cells (5 × 10^5^) were added to each well. Twenty-four hours after transfection lysates were prepared by aspirating the media, washing the cells once with PBS, then adding 45 μL per well of 1 × Passive Lysis Buffer (Promega; Madison, WI). Luciferase assays were performed using 20 μL lysate and the Dual Luciferase Assay Kit (Promega; Madison, WI) on a GloMax Multi-detection System (Promega; Madison, WI). To normalize firefly luciferase values based on the number of living transfected cells, firefly luciferase values were divided by Renilla luciferase values for each sample. Normalized firefly luciferase values were compared using a one-way ANOVA and Bonferroni's multiple comparison test, as implemented in GraphPad v5.04.

For metal specificity, cells were transfected as above and simultaneously treated with the indicated concentration of FAC, CuSO_4_, or ZnSO_4_. For endocytosis experiments, Aag2 cells were seeded in a 96-well plate at 50,000 cells/well. After 24 h, cells were transfected as above except with 18 ng pGL3-AaLCH-FF + 6 ng pSLfa-PUb-RL. After another 24 h, cells were treated with 80 μM Dynasore, 75 μM EPIA, 100 μM Deferoxamine or DMSO alone with or without 100 μM FAC. Dynasore-treatment was stopped at 1.5 h to prevent cell death (media was replaced) and cells for all treatments were harvested after 8 h with luciferase assays and statistical analysis performed as above.

### Bioinformatics and phylogenetic analyses

Amino acid sequences for mosquito (Vectorbase.org), Drosophila (Flybase.org) or human (Genbank) ZIP (SLC39), ZnT (SLC30), and NRAMP (SLC11) genes were downloaded from the respective databases and aligned using ClustalW (Larkin et al., 2007) in MEGA6 (Tamura et al., 2013). Refseq numbers for each sequence are provided in Supplementary Table 2. Multiple sequence alignments were used to infer phylogenetic trees using the Neighbor-joining method in MEGA6, with the pairwise deletion option and the bootstrap method for branch support (1,000 replicates). To search for Mvl/NRAMP orthologs in culicines, the *D. melanogaster* and *An. gambiae* NRAMP protein sequences were used as queries using either NCBI blastp (refseq) or Vectorbase blastp [*A. aegypti* peptides, AaegL3.5; *A. albopictus* peptides, AaloF1.2; C6/36 cells, NCBI-101; Cx. Quinquefasciatus peptides, CpipJ2.4] and tblastn [*A. aegypti* ESTs; AaegL3.5 geneset, AaegL3 contigs, AaegL3 scaffolds; C636 cells transcripts, NCBI-101; *A. albopictus* assembled transcriptome TSA:GAPW00000000.1; AaloF1.2 transcripts, AaloF1 contigs, AaloF1 scaffolds; CpipJ2 contigs, CpipJ2 scaffolds, CpipJ2.4 geneset). A single EST identified from *A. aegypti* larvae was used as a query in NCBI blastx. For all blast searches, Max *e*-value = 10, Word size = 3, Scoring Matrix = Blosum62 and the number of results per query was 50.

### Mosquitoes

For all experiments, *A. aegypti* (Liverpool strain) was maintained in an insectary in humidified chambers at 28°C, 70% RH and 14H:10H (L:D) cycle. The mosquito colony was maintained on defibrinated sheep blood (Colorado Serum Company, Denver CO) using an artificial feeding system.

### Organ-specific expression

To examine organ-specific transcript expression, midguts (Mg), Malpighian tubules (MT), ovaries (Ov) and carcasses (whole body without Mg, MT and Ov) were dissected from female mosquitoes in 1 × PBS and transferred immediately into 1.5-mL tubes containing TRIzol reagent (Thermo Fisher). Samples were taken from sugar-fed (5 days after eclosion), 6 h post-bloodmeal (PBM) and 24 h PBM for three biological replicates.

To investigate effects of free iron on AaeZIP13 expression, female mosquitoes were fed with an artificial meal (AM) containing 150 mM NaCl, 20 mM NaHCO_3_, 1 mM ATP and 1% low melt temperature agarose supplemented with 5 mM of FAC using an artificial feeding system. At 24 h after feeding, midguts were dissected for qRT-PCR analysis (pools of 20 individuals for each of three biological replicates).

RNA was isolated using the TRIzol method following the manufacturer's protocol (Thermo Fisher), and the total RNA was treated with RNase-free DNase (TURBO DNAfree kit: Thermo Fisher). Isolated RNA was quantified on a SpectraMax (Molecular Devices, Sunnyvale, CA). One microgram of RNA from each sample was used to synthesize cDNA using anchored oligo d(T) primers (d(T)_20_-VN) with MultiScribe reverse transcriptase (Applied Biosciences) at 42°C for 2 h and reverse transcriptase was inactivated at 65°C for 20 min. cDNA was diluted to 1/50 in nuclease-free H_2_O, which was used for qRT-PCR analysis.

Quantitative real-time PCR (qRT-PCR) was performed on CFX69 Touch Real-Time PCR Detection System (Bio Rad), using PerfeCTa qPCR FastMix II (QuantaBio). Primers listed in Supplementary Table 1 were designed using Primer3 server (version 4.0.0) (Untergasser et al., 2012) or PrimerSelect (DNASTAR), which amplify 107–247 bp fragments of cDNA and amplification efficiency (E) was empirically verified to be 0.9–1.0. Reactions were performed in 15 μL with 200 nM each primer and 3.75 μL of 1/50 diluted cDNA in triplicates. All reactions were performed with 30 s at 95°C, followed by 45 cycles of 5 s at 95°C, 15 s at 60°C and 10 s at 72°C, and melt curve analysis at 70–95°C. Expression was calculated relative to the reference housekeeping gene rpS7 by the dCt method (Liu and Saint, 2002). C_q_ values as well as calculated efficiencies for all primer sets/samples are presented in Supplementary Table 3.

### Mosquito RNAi

To knockdown the expression of each candidate iron transporter in whole mosquitoes, female *A. aegypti* (2–3 days after eclosion) were injected with 1 μg of dsRNA using a Nanoject II microinjector (Drummond) with a needle drawn from glass capillary and were kept at 28°C rearing chamber for indicated time until each experiment was performed. Control group of mosquitoes was injected with 1 μg of dsRNA against exogenous EGFP sequence.

#### Semi-quantitative intracellular free Fe^2+^ estimation by calcein AM and fecundity/fertility test

To determine the relative cytoplasmic iron content of dsRNA-injected mosquitoes, injected individuals were fed on defibrinated sheep blood (Colorado Serum Company) at 3 days post injection. From a part of each group (10–15 individuals), midguts and ovaries at 24 h PBM were dissected and treated with 2.5 μM of calcein AM (Thermo-Fisher) in 1 × PBS at room temperature on an orbital shaker. After 1 h incubation, calcein AM solution was removed and replaced with 1 × PBS. Fluorescence images of the organs were captured at Ex: 450–490/Em: 500–550 with the same exposure time and gain setting within the same organ for each experiment using Leica M165 FC stereomicroscope equipped with DFC3000C digital camera. The images were analyzed by ImageJ (Schneider et al., 2012) for pixel intensity using manual polygon tool. Calculated values were compared using a Students two-tailed *t*-test as implemented in GraphPad v5.04.

To measure the effect of dsRNA treatment on fertility, the remaining individuals were kept until 72 h PBM and individually placed in a well of a 24-well plate with 2% agarose on the bottom as a wet surface for egg laying for 24 h. After laying eggs, each well was photographed by a digital camera (Olympus TG-4, microscope mode) to count eggs and water was added to the wells. After additional 5 days, each well of the plate, now with hatched larvae was photographed and the hatched larvae were counted. The number of eggs/larvae were compared with the control group using a Students two-tailed *t*-test as implemented in GraphPad v5.04.

### Chemicals

Deferoxamine (mesylate) [DFO], 3-hydroxy-2-[(3,4-dihydroxyphenyl)methylene]hydrazide-2-naphthalenecarboxylic acid [Dynasore] and 5-(N-ethyl-N-isopropyl)-Amiloride [EIPA] were obtained from the Cayman Chemical Company. Iron (II) sulfate heptahydrate (F8633), ferric citrate (F3388), copper (II) sulfate pentahydrate (C8027), and zinc sulfate heptahydrate (Z0251) were obtained from Sigma-Aldrich. Ferric ammonium citrate (40600001) was obtained from Bioworld. Calcein acetoxymethyl (AM) was obtained from Life Technologies.

## Results

**Figure 1:**
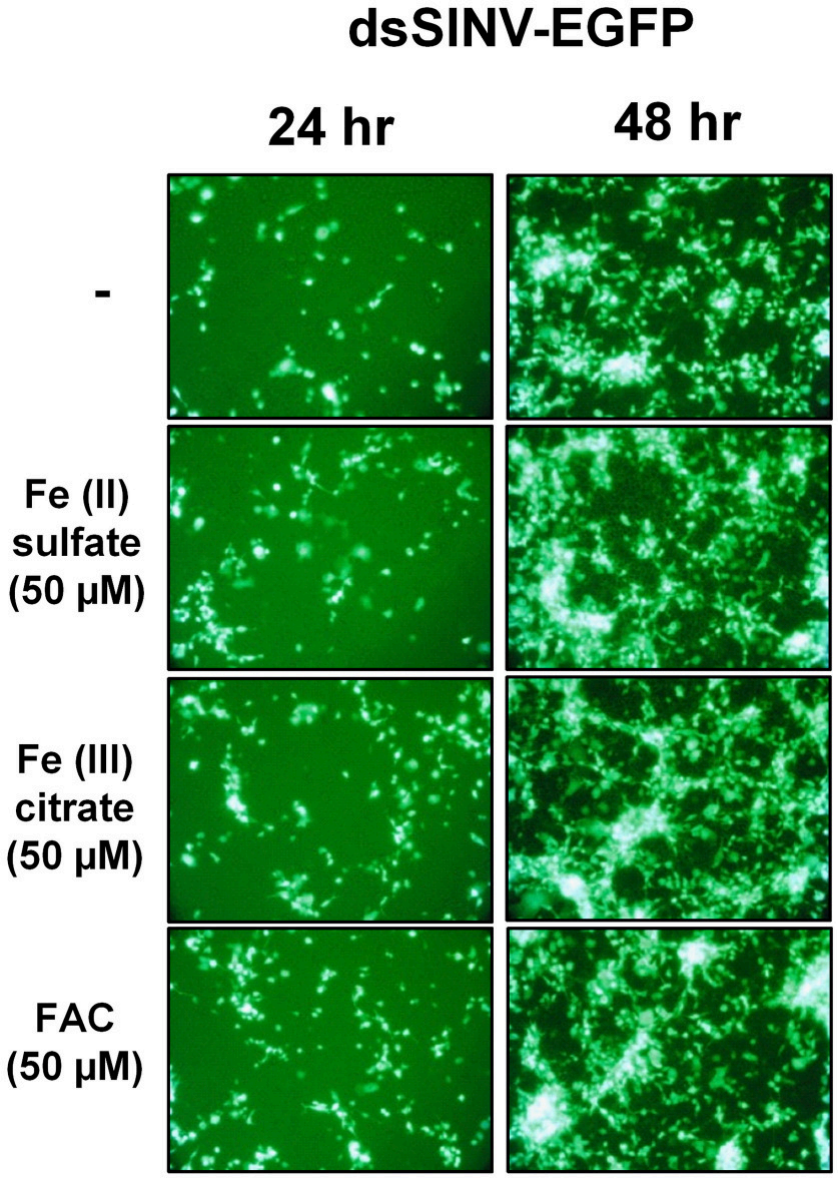
Treatment with Fe^2+^ or Fe^3+^ does not interfere with SINV replication in *A. aegypti* cells. Aag2 cells were pre-treated with the indicated Fe compound and then challenged with a recombinant SINV expressing EGFP. Cells were photographed at 24 and 48 h post infection.

Rose et al. (2011) reported that the iron importer NRAMP (Mvl) serves as a receptor for SINV entry into *Drosophila*, vertebrate and even *A. aegypti* mosquito cells. In particular, Rose et al. (2011) suggested that treating *Drosophila* or *A. aegypti* cultured cells with 32 or 160 μM Fe^3+^ was sufficient to substantially inhibit the entry of SINV, due to a presumed down-regulation of NRAMP in response to Fe overload. We sought to confirm these findings, as a bioinformatic search of annotated genes, scaffolds, contigs, and available ESTs using either the *Drosophila* Mvl/NRAMP or *Anopheles gambiae* Mvl/NRAMP protein sequences failed to reveal an NRAMP ortholog in any of the sequenced culicine mosquito genomes (*A. aegypti, A. albopictus, Cx. quinquefasciatus)* (Table 1 and Supplementary Table 4). [Note: a single EST described as coming from *A. aegypti* larvae was identified with an *e*-value of 6e-28 and 49% percent identity to *An. gambiae* NRAMP, but this EST was found to be 93% identical to NRAMP from Barley, suggesting it as a contaminant of the larval diet. No other *e*-value was less than 0.4]. To replicate the experimental protocol reported by Rose et al. (2011), we treated *A. aegypti* Aag2 cells with 50 μM of reduced (Fe II sulfate) or oxidized (Fe III citrate; ferric ammonium citrate FAC) iron and challenged the cells with a recombinant SINV expressing EGFP. We observed no difference in the number or brightness of SINV-EGFP infected cells at 24 or 48 h (Figure 1). We conclude that NRAMP is not likely a receptor for SINV in *A. aegypti*, or for any culicine mosquitoes that serve as a natural vector of this virus. As NRAMP is a highly conserved (when present, anyway) importer of dietary iron, and its absence in culicine mosquitoes suggests that alternative methods for the import of molecular iron must have evolved to compensate for the loss of this gene. Thus, we turned our attention to the identification of alternative iron transporters in *A. aegypti*.

**Table 1:** Blastp-based search of mosquito proteins using the Malvolio (*D. melanogaster* NRAMP ortholog) protein sequence as a query.

**Hit**	**Description**	**Query**	**Aln length**	***E*-value**	**Score**	**Identity**
**AGAP012464-PA**	**Natural resistance-associated macrophage protein**	**Dmel_Mvl-PB**	**373**	**4E-163**	**1650**	**0.612**
***An. gambiae***						
AGAP010308-PA	Nucleolar complex protein 3	Dmel_Mvl-PB	56	1.8	91	0.25
AGAP013324-PA	Putative G-protein coupled receptor GPCR	Dmel_Mvl-PB	51	3	89	0.308
***Cu. quinquefasciatus***						
CPIJ005183-PA	Organic anion transporter, putative	Dmel_Mvl-PB	131	3.6	88	0.246
CPIJ010125-PA	Conserved hypothetical protein	Dmel_Mvl-PB	53	5.7	86	0.321
CPIJ001796-PA	Conserved hypothetical protein	Dmel_Mvl-PB	55	7.3	85	0.273
CPIJ014837-PA	Serine/threonine protein kinase lats	Dmel_Mvl-PB	34	6.5	85	0.382
***A. aegypti***						
AAEL013826-PA	Serine/threonine protein kinase lats	Dmel_Mvl-PB	40	2.4	90	0.4
AAEL004986-PA	Smg-7 (suppressor with morphological effect on genitalia protein 7)	Dmel_Mvl-PB	51	5	86	0.25
AAEL010052-PA		Dmel_Mvl-PB	20	2.8	86	0.4
AAEL012774-PA	Protease m1 zinc metalloprotease	Dmel_Mvl-PB	49	7.8	84	0.204
***A. albopictus***						
AALF008030-PA	Serine/threonine protein kinase lats	Dmel_Mvl-PB	40	0.51	98	0.4
AALF005921-PA		Dmel_Mvl-PB	42	0.89	92	0.468
AALF022221-PA		Dmel_Mvl-PB	42	0.89	92	0.468
AALF027921-PA	GPCR Leukokinin Family	Dmel_Mvl-PB	47	4.2	87	0.319
AALF021769-PA		Dmel_Mvl-PB	49	9.4	83	0.347
AALF012982-PA		Dmel_Mvl-PB	86	8.7	82	0.25
AALF026370-PA		Dmel_Mvl-PB	49	9	80	0.327

**Figure 2:**
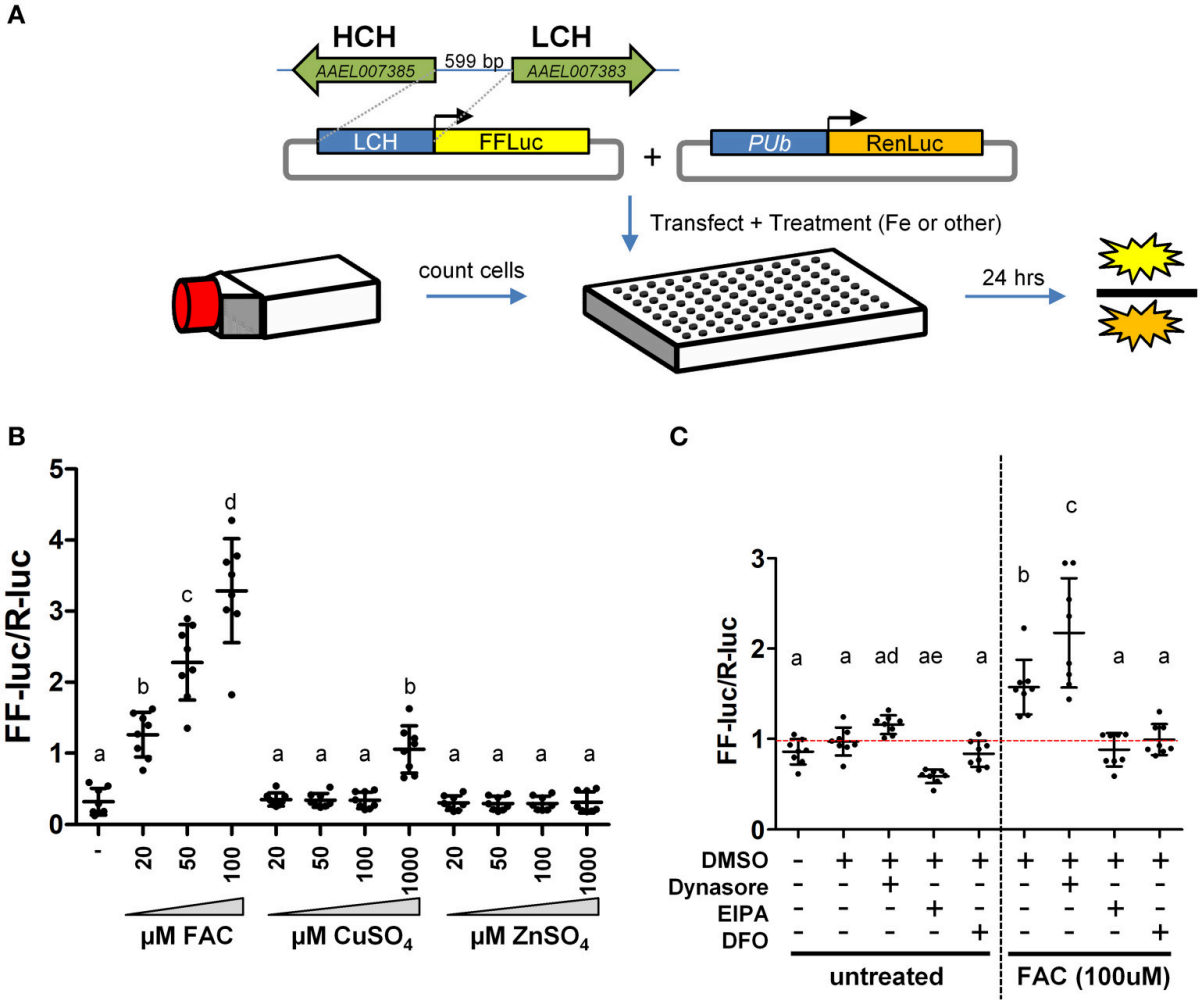
A luciferase-based sensor induced by iron. **(A)** Schematic representation of the experimental workflow. *A. aegypti* cells were counted, mixed with transfection reagent/plasmid DNA and seeded into 96 cell plates with the indicated metal included in the cell culture medium. **(B)** Ratio of firefly luciferase (FF-luc) to *Renilla* luciferase (R-luc) in Aag2 cells after treatment with the indicated concentration of Fe, Cu or Zn. **(C)** Ratio of FF-luc to R-luc in Aag2 cells treated with Dynasore (80 μM, 1.5 h), EIPA (75 μM, 8 h), DFO (100 μM, 8 h). Luciferase values were determined after 8 h for all samples. For **B** + **C**, mean (horizontal line) and standard deviation (error bars) are indicated; lower case letter designations indicate statistically different groups following ANOVA (*P* < 0.0001) and Bonferroni's multiple comparison test (*P* < 0.01) (GraphPad v5.04). Red dotted line indicates the mean of the DMSO untreated sample.

Two large gene families, the ZIPs and ZnTs, are known to contain members that transport iron across membranes; *A. aegypti* encodes 10 members of the ZIP family and 8 ZnTs. In order to identify those that might be involved in the transport of iron, we constructed a luciferase-based sensor whereby the luciferase coding sequence was placed under the control of a promoter derived from the ferritin light chain gene (Figure 2A), a gene previously shown to be induced by iron treatment (Pham and Chavez, 2005). Following transfection of the plasmid into *A. aegypti* cells, we sought to establish the specificity of the reporter. Cells were incubated with increasing concentrations of Fe, Cu, or Zn and luciferase activity determined. We observed strong activation only in the presence of Fe (Figure 2B). Further experiments demonstrated that both reduced (Fe^2+^) or oxidized (Fe^3+^) iron served equally well to activate the reporter, and that at least 8 h was required to observe activation (Supplementary Figure 1). Next, we sought to determine whether endocytic pathways are involved in the uptake of free iron, as opposed to transport at the cell surface. *A. aegypti* cells were treated with the dynamin inhibitor Dynasore [inhibits both endo- and exo-cytosis; (Macia et al., 2006)] or the Na^+^/H^+^ exchanger inhibitor EIPA [inhibits macropinocytosis; (Fretz et al., 2006)]. As a control, cells were also treated with the iron chelator deferoxamine (DFO). In the presence of FAC, inhibiting dynamin filaments substantially increased reporter activity in the cells, while inhibiting Na^+^/H^+^ exchange decreased induction of ferritin. These experiments suggest that macro-pinocytosis may be used during iron import, and that dynamin-dependent processes such as exocytosis may be critical to reducing iron levels through export (Figure 2C).

**Figure 3:**
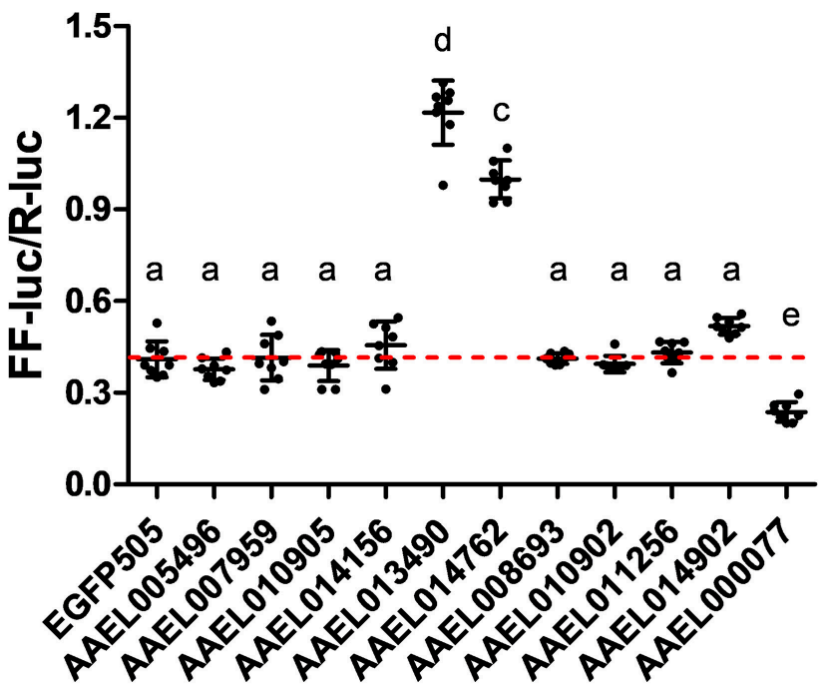
Knockdown of ZIP or ZnT genes alters ferritin induction in *A. aegypti* cells. Ratio of firefly luciferase (FF-luc) to *Renilla* luciferase (R-luc) in Aag2 cells after treatment with the indicated dsRNA. Mean (horizontal line) and standard deviation (error bars) are indicated; lower case letter designations indicate statistically different groups following ANOVA (*P* < 0.0001) and Bonferroni's multiple comparison test (*P* < 0.01) (GraphPad v5.04). Red dotted line indicates the mean of the EGFP505 dsRNA treated sample.

**Figure 4:**
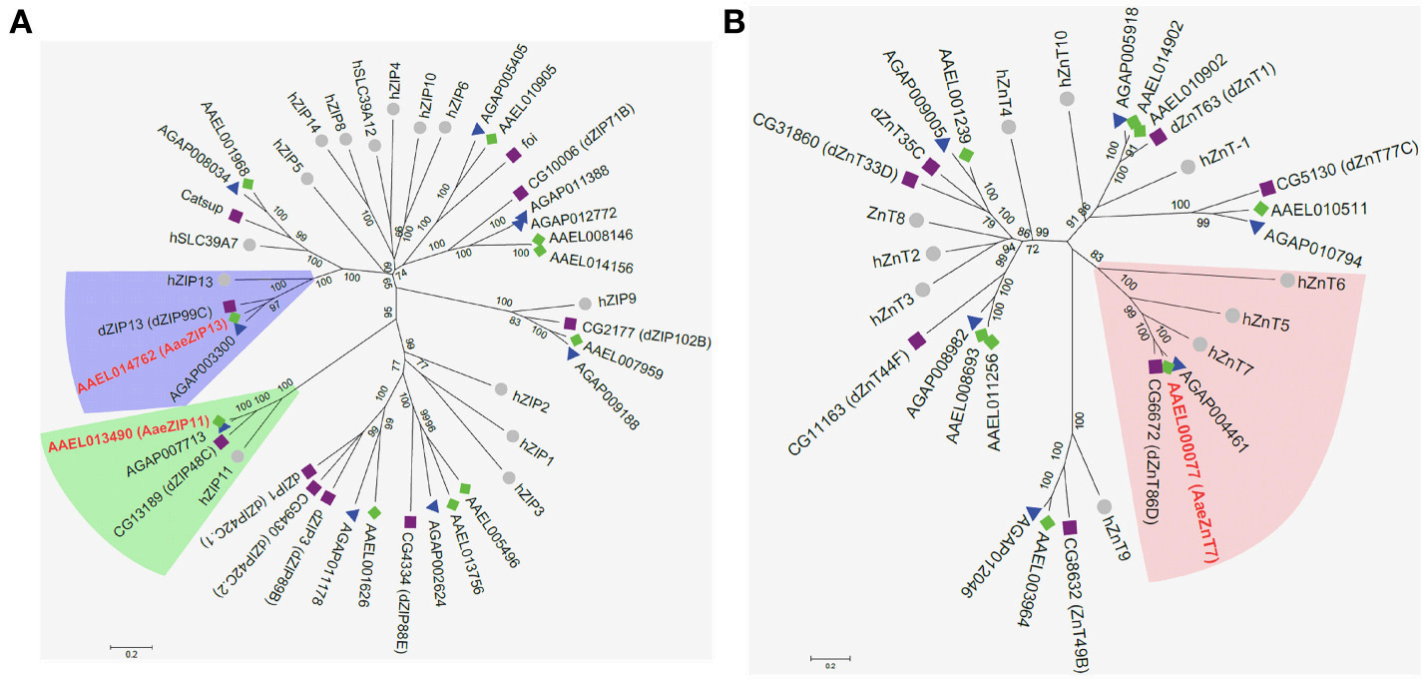
Phylogenetic analysis of *A. aegypti* ZIP and ZnT genes. Neighbor-joining trees were produced in MEGA 6 following ClustalW alignment of ZIP **(A)** or ZnT **(B)** protein sequences from humans (•), *D. melanogaster* (■), and mosquitoes [*An. gambiae* (▴) and *A. aegypti* (♦)]. Clades containing Fe-sensitive transporters identified in our assay are highlighted (ZIP13, blue; ZIP11, green; ZnT7, peach). Putative *A. aegypti* iron transporters are in red-bold letters.

Having confirmed that our luciferase reporter can detect changes in iron-dependent ferritin induction in mosquito cells, we designed double-stranded RNAs for each of the *A. aegypti* ZIPs and ZnTs and performed RNAi knockdown prior to transfection and measurement of the luciferase reporter. While a few genes produced small, but significant changes in a subset of experiments (Supplementary Figure 2), we repeatedly observed only three genes (AAEL014762, AAEL013490, AAEL000077) that when knocked down resulted in substantial changes in luciferase activity (Figure 3). Of these, two are members of the ZIP family (AAEL014762 and AAEL013490 are 1:1 orthologs of the fly and human ZIP13 and ZIP11 genes) and one a member of the ZnT family (AAEL000077, an ortholog of the fly ZnT86D and human ZnT7 genes) (Figures 4A,B).

**Figure 5:**
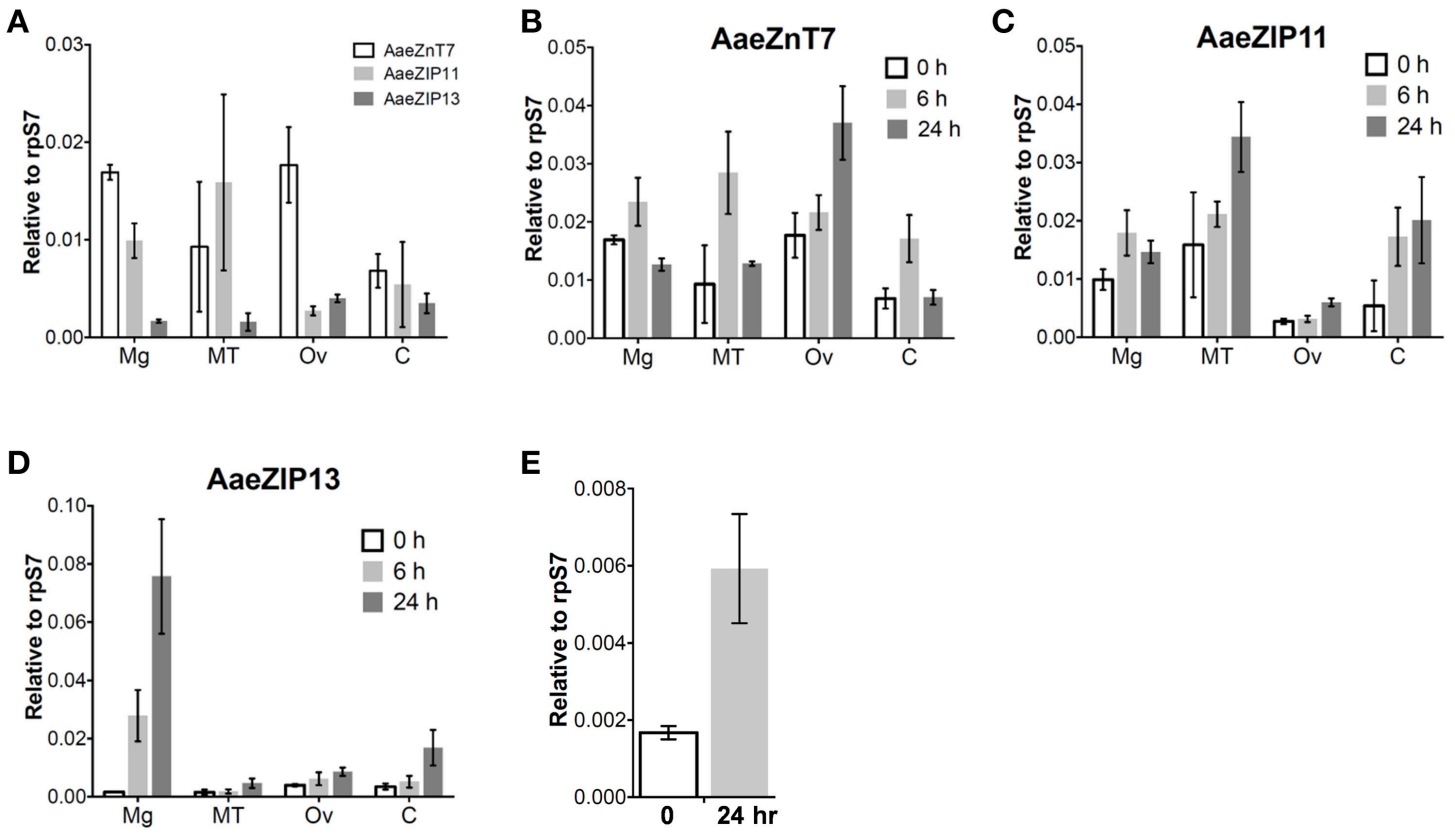
Organ-specific transcript expression of candidate iron transporter genes by qRT-PCR. **(A)** Transcript expression in organs dissected from sugar-fed females (before bloodfeeding). Transcript expression of AaeZnT7 **(B)**, AaeZIP11 **(C)**, and AaeZIP13 **(D)** in organs dissected at the indicated times post-bloodmeal; graphs show mean ± standard deviation. **(E)** AaeZIP13 expression 24 h after Fe-only artificial meal. Values in 0 h in panel **(B–E)** are the same as values in panel **(A)**. Mg, midgut; MT, Malpighian tubules; Ov, ovaries; C, carcass (whole body without Mg, MT, and Ov). *N* = 3 pools, with 20 individuals per pool.

We next investigated the organ-specific expression of each candidate iron transporter gene by quantitative real-time PCR (qRT-PCR) in the midgut (Mg), ovaries (Ov), and Malpighian tubules (MT) of the adult mosquito. Prior to bloodfeeding, AaeZnT7 is enriched in the Mg and Ov, AaeZIP11 is enriched in Mg and MT, and AaeZIP13 is enriched in Ov with the overall lowest expression (Figure 5A). Following a bloodmeal, the expression of all three transcripts increased. The most prominent up-regulation was observed for AaeZIP13 in the midgut, with an almost 45-fold increase in transcript levels compared to the resting state at 24 h PBM, while other genes merely increased up to 2-fold (Figures 5B–D). We further examined whether this up-regulation of AaeZIP13 upon bloodmeal is a specific response to free iron in the bloodmeal by feeding an artificial meal (AM) containing salt and FAC. AaeZIP13 expression was significantly up-regulated 24 h after the AM although the extent of up-regulation was much less than a real bloodmeal (Figure 5E). These results indicate that AaeZIP13 exhibits a strong response to the components in blood or blood feeding, which is likely related in iron transport during blood meal digestion.

**Figure 6:**
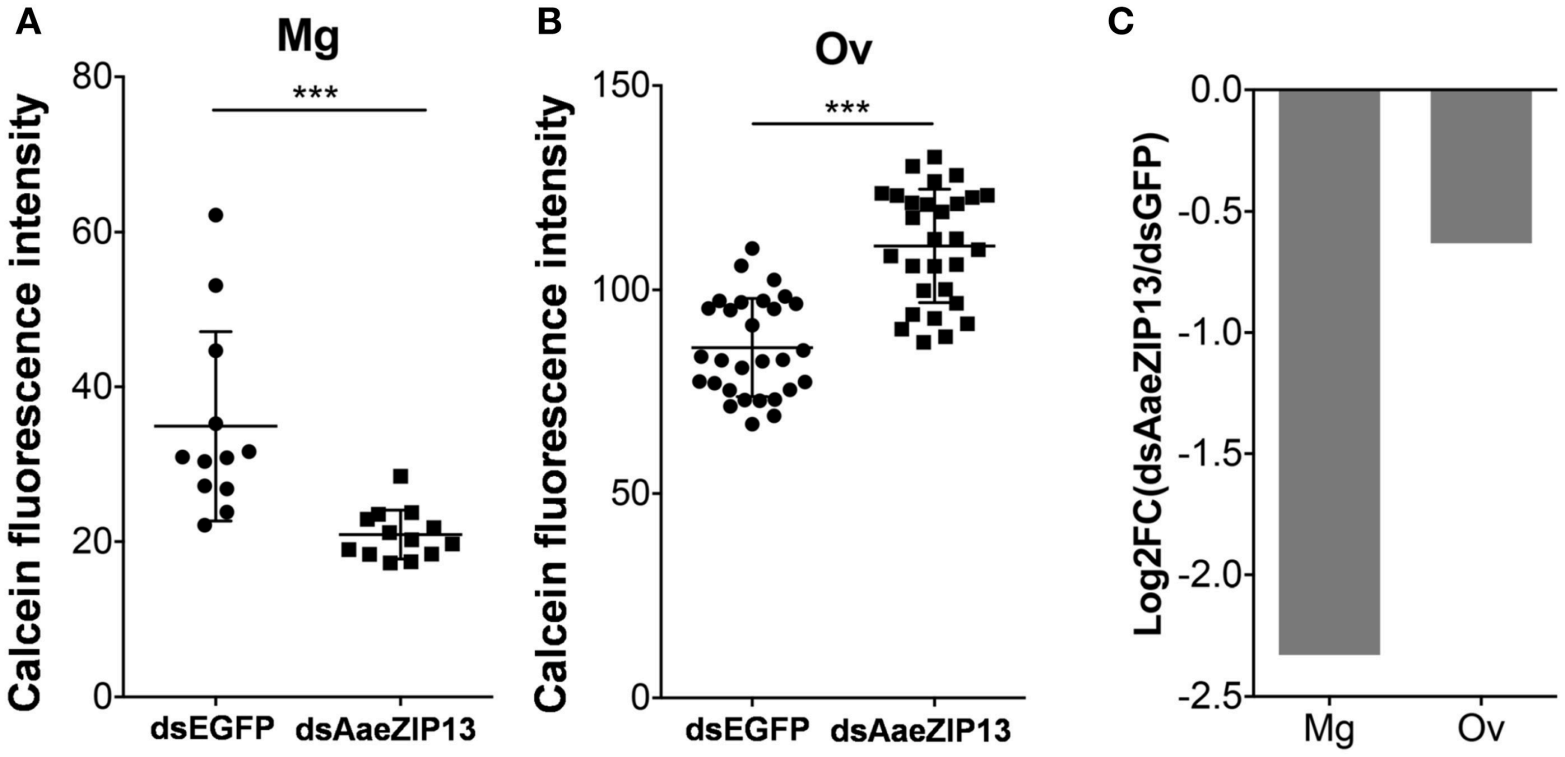
Iron accumulation is disrupted following AaeZIP knockdown. Calcein fluorescence at 24 h post blood feeding in the midgut **(A)** and ovaries **(B)** from AaeZIP13 knockdown and control (dsEGFP) mosquitoes. For **(A)**, each dot represents a single posterior midgut; **(B)** each dot represents a lobe of ovary; lines indicate mean and standard deviation. Figures are representative of 5 independent experiments. ^***^ indicates *p* < 0.001 by unpaired two-sided Student's *t*- test. **(C)** Knockdown efficiency of dsRNA injection at 4 day post dsRNA injection/24 h post blood feeding. Graph shows Log_2_ transformed fold change (dsAaeZIP13/dsEGFP) values of qRT-PCR results (normalized to rpS7 expression). Mg, midgut; Ov, ovaries.

**Figure 7:**
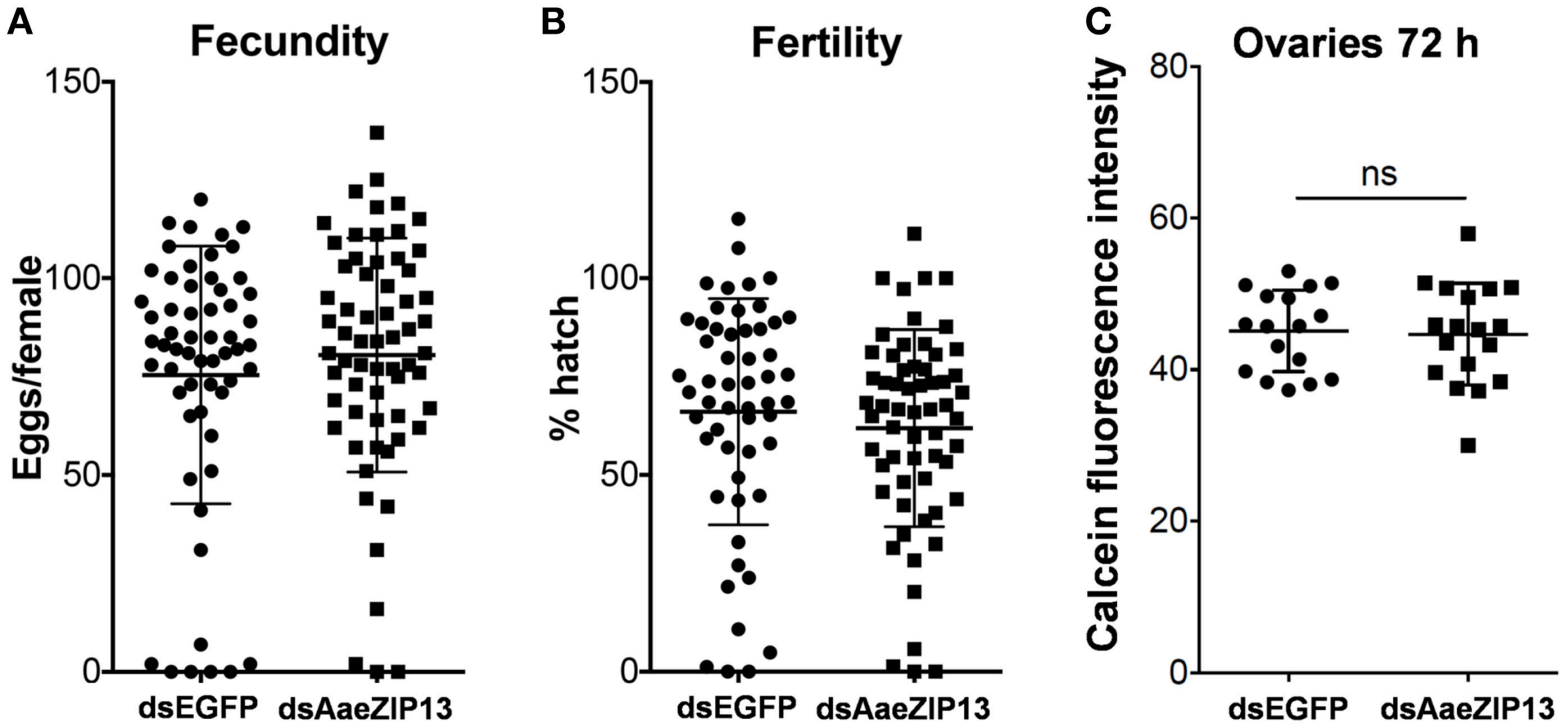
Fecundity (egg number) and fertility (hatch rate) from AaeZIP13 knockdown and control (GFP) mosquitoes. **(A)** Fecundity (egg number per female); **(B)** Fertility (hatch rate per laid egg batch). Lines indicate mean ± standard deviation. Means are not significant by unpaired, two-sided *t*-test. **(C)** Calcein fluorescence at 72 h post blood feeding in the ovaries from AaeZIP13 knockdown and control (EGFP) mosquitoes.

We therefore sought to determine a physiological role of AaeZIP13 during blood-meal digestion and reproduction following RNAi-based gene silencing. Female mosquitoes were injected with dsRNA against AaeZIP13 or control (EGFP) and were fed on blood at 3 days post injection (dpi); midguts and ovaries were dissected for calcein AM treatment at 24 h PBM. Calcein AM freely diffuses across the plasma membrane and is converted to fluorescent calcein in living cells; the fluorescence is quenched when it is bound to iron (Fe^2+^). Knockdown of AaeZIP13 resulted in a significant decrease in calcein fluorescence in the midgut, signifying an increase in cytoplasmic iron content. Conversely, we observed an increase in calcein fluorescence in the ovaries at 24 h PBM suggesting less iron accumulation at this time (Figures 6A,B). We also confirmed that dsRNA effectively reduced transcript levels in the Mg and to the lesser extent in the Ov at 24 h PBM (Figure 6C). As iron levels in the ovaries were altered upon AaeZip13 depletion, we further investigated the impact of AaeZIP13 gene silencing on fecundity and fertility. We detected no difference in fecundity (egg number per female) and fertility (hatch rate) (Figures 7A,B). Likewise, iron content in the ovaries following AaeZip13 dsRNA treatment at 72 h PBM was indistinguishable from the control (Figure 7C).

## Discussion

Contrary to the observation made by Rose et al. (2011), we found that ionic iron had no effect on SINV infection in *A. aegypti* Aag2 cells. While it is possible that treatment with excess iron did not modulate NRAMP expression in mosquito cells, no homolog of NRAMP was found in the published genomes of three culicine mosquitoes. We conclude from these results that culicine mosquitoes, including *A. aegypti*, appear to lack NRAMP, the best studied importer of dietary iron to date. This raised the question of how *A. aegypti* acquire dietary iron from the gut lumen and led us to search for alternative iron transporters in this mosquito. Taking advantage of a ferritin gene known to be strongly induced by cytoplasmic iron levels, we constructed a luciferase-based iron-specific sensor-reporter construct. Though we did not test the response of this sensor to every divalent metal possible, Zn, and to a much lesser extent Cu, were some of the only metals likely to be present at physiologically relevant concentrations in our cell culture media. Inhibition of general export/import processes confirmed that the acquisition of iron is indeed an active process requiring endocytosis. This suggests that potential iron importers do not act to a large extent on the cell surface but instead in endocytic vesicles. Using our sensor-reporter, we screened genes in families with potential zinc and iron transporting function (ZIP and ZnT families), and identified three genes as candidate iron transporters; orthologs of human ZnT7 (AAEL000077), ZIP11 (AAEL013490), and ZIP13 (AAEL014762).

In *D. melanogaster* the ZnT7 ortholog (ZnT86D; CG6672) was described as a zinc transporter that localizes in ER/Golgi for zinc storage (Richards and Burke, 2016). When ZnT7 was overexpressed in the midgut or systemically suppressed, *Drosophila* exhibited lethality in the larval stage (Lye et al., 2012). Results of our qRT-PCR indicated that the transcript of AaeZnT7 is enriched in the midgut and ovaries, indicating the potential for involvement in the acquisition of nutrients from the blood meal. Upon bloodfeeding, AaeZnT7 transcripts increased in the Malpighian tubules and midgut at 6 h PBM, returning to resting level at 24 h PBM with a corresponding increase in transcripts at 24 h PBM in the ovaries. This pattern suggests several potential roles for AaeZnT7: importation of iron from the blood meal into the midgut epithethelial cells, and/or importation of iron from the hemolymph into the Malpighian tubules (for excretion of excess) and ovaries (for nutrition).

The *Drosophila* ZIP11 ortholog (ZIP48C; CG13189) is highly enriched in the Malpighian tubules (Chintapalli et al., 2010), but functional characterization data is lacking in this species. However, its mammalian counterpart has been characterized as a zinc transporter, which is most closely related to prokaryotic zinc transporters (Yu et al., 2013). AaeZIP11 transcripts were also found to be enriched in the Malpighian tubules, particularly following a bloodmeal, suggesting a potential role in excretion. As the key organ for insect excretion and detoxification, Malpighian tubules may play a critical role in excretion of excess iron accumulated in the hemolymph (Folwell et al., 2006; Beyenbach et al., 2010; Li et al., 2017). As a candidate exporter, AaeZIP11 may function in concert with AaeZnT7 (candidate importer) to transfer iron from the hemolymph to the MT lumen for excretion, and we are actively investigating this possibility.

While no previous role in iron transport has been reported for ZnT7 or ZIP11, the ortholog of ZIP13 in *D. melanogaster* (dZIP13/dZIP99C: CG7816) has recently been characterized as an iron transporter, critical to the export of iron from the cytoplasm of midgut epithelial cells into the ER lumen where loading into ferritin occurs (Xiao et al., 2014). In contrast to the minor blood-meal induced transcriptional changes we observed for AaeZIP11 and AaeZnT7, AaeZIP13 transcripts increased about 45 times that at the resting state after blood feeding, most prominently in the midgut. Iron-only AM feeding resulted in up-regulation of AaeZIP13 transcript in the midgut, which supports our hypothesis. The much less up-regulation of AaeZIP13 than with a real blood in this assay could be due to the lack of other complex signals induced by real blood meal (proteins, amino acids, heme, etc.) and associated hormonal controls. RNAi-mediated gene knockdown resulted in reduced calcein fluorescence, and thus increased cytoplasmic iron content in the midgut consistent with a role for AaeZIP13 in iron export from this tissue. This phenotype is similar to *Drosophila* dZIP13, whose knockdown resulted in reduction of iron in the body but accumulation of iron in the midgut (Xiao et al., 2014). This similarity suggests that the function of ZIP13 is conserved in Diptera. Retention of iron in the midgut delayed, but did not prevent, iron accumulation in the ovaries. This delayed iron accumulation in the ovaries had no detectable impact on fecundity and fertility of female mosquitoes. This could be due to residual ZIP13 activity in the midgut due to incomplete suppression of AaeZIP13 transcripts by RNAi. Alternatively, there may be other iron transporters serving in a redundant fashion to ensure that sufficient iron reaches the ovaries. The development of CRISPR/Cas9-based knockout strains may provide clarity in this regard, and these experiments are currently under investigation.

The apparent lack of a Mvl/NRAMP transporter in culicine mosquitoes suggests an alternative mode of dietary iron acquisition in these species. RNAi-based screening of the ZIP and ZnT families revealed several candidate iron transporters, but none that fit the profile anticipated of a midgut-associated dietary importer (though more evidence is clearly needed for AaeZnT7). One or more such transporters are presumed to be present, as it is known that human transferrin-bound iron is efficiently absorbed into the body of *A. aegypti* during bloodfeeding (Zhou et al., 2007). Moreover, gene(s) functionally replacing NRAMP may also play roles in mosquito behavior because highly conserved Mvl homologs are related to feeding behavior in *D. melanogaster* (Sovik et al., 2017), foraging behavior of honey bee (*Apis mellifera*) (Ben-Shahar et al., 2004), invading-reproducing behavior in its parasite verroa mite (*Verroa destructor*) (Cabrera et al., 2014) and potential correlation to parental care and social interaction of a carrion beetle, *Nicrophorus vespilloides* (Mehlferber et al., 2017). Since Mvl also transports copper, such gene(s) replacing NRAMP might as well have similar function (Southon et al., 2008; Navarro and Schneuwly, 2017). We suspect that another transporter family has been recruited to perform this function during culicine evolution, and are in the process of expanding our RNAi-based screen to identify additional candidates. The iron-specific sensor-reporter system we have developed is thus a valuable tool to detect iron-induced regulatory changes in mosquito cells and aid in the identification of genes and molecules involved in iron homeostasis and transport in this important disease vector mosquito.

## Author contributions

HT, MA, KM, and ZA: design of the work; HT, MA, and ZA: performed experiments; HT and ZA: drafting manuscript; HT, MA, KM and ZA: approved the final version.

### Conflict of interest statement

The authors declare that the research was conducted in the absence of any commercial or financial relationships that could be construed as a potential conflict of interest.
