# Supplementary material for: Identification of Candidate Iron Transporters From the ZIP/ZnT Gene Families in the Mosquito Aedes aegypti
Source: Front Physiol. 2018 Apr 12;9:380. doi: 10.3389/fphys.2018.00380 (PMC5906682; doi:10.3389/fphys.2018.00380)
Supplement: Supplementary file 1 [file DataSheet1.DOCX]

Supplementary Material

Identification and characterization of iron transporters in the mosquito *Aedes aegypti*

Hitoshi Tsujimoto^1^, Michelle A. E. Anderson^2^, Kevin M. Myles^1^ and Zach N. Adelman^1*^

^1^ Department of Entomology and Agrilife Research, Texas A&M University, College Station, TX 77843

^2^ Department of Entomology and Fralin Life Science Institute, Virginia Tech, Blacksburg, VA 24061.

* Correspondence: Zach N. Adelman: zachadel@tamu.edu

# Supplementary Data

Supplementary Material should be uploaded separately on submission. Please include any supplementary data, figures and/or tables.

Supplementary material is not typeset so please ensure that all information is clearly presented, the appropriate caption is included in the file and not in the manuscript, and that the style conforms to the rest of the article.

# Supplementary Figures and Tables

For more information on Supplementary Material and for details on the different file types accepted, please see [here](http://home.frontiersin.org/about/author-guidelines#SupplementaryMaterial).

## Supplementary Figures

**Supplementary Figure 1.** **Activation of luciferase-based sensor of iron stress based on Fe source and time**. Ratio of Firefly (FL) to Renilla (RL) in Aag2 cells after treatment with the indicated concentration (in µM)/type of Fe (**A**) or after the indicated time (**B**). Mean (horizontal line) and standard deviation (error bars) are indicated; lower case letter designations indicate statistically different groups following ANOVA and Bonferroni’s multiple comparison test (GraphPad v5.04).

**Supplementary Figure 2.** **Activation of luciferase-based sensor of iron stress following RNAi of ZIP or ZnT genes**. Ratio of Firefly (FF-luc) to Renilla (R-luc) in Aag2 cells after treatment with the indicated dsRNA. Mean (horizontal line) and standard deviation (error bars) are indicated; lower case letter designations indicate statistically different groups following ANOVA and Bonferroni’s multiple comparison test (GraphPad v5.04). Red dotted line indicates the mean of the EGFP dsRNA (GFP505) treated sample. Shaded boxes highlight AaeZnt7 (AAEL000077), AaeZIP11 (AAEL013490) and AaeZIP13 (AAEL014762).

| **Gene ID/accession #** | **Forward** | **Reverse** | **class** | **purpose** |
| --- | --- | --- | --- | --- |
| AAEL007383 | TTTTAAGCTTCCACCTCTATCGGTGTCCGGTACTAGTCTGA | TTTTCCATGGTAACTCGTTACTGCAAGATCACAATTACACACACTAG |  | FrLCH promoter cloning |
| AAEL000077 | **TAATACGACTCACTATAGG**AGATTATGCGCGGAGTCTTTCTG | *GGAAAAAAA*GCGGCATAGTCGAGCTGGATGTAG | ZnT | dsRNA temp |
| AAEL001239 | **TAATACGACTCACTATAGG**GCCATGGCCACAGTCACTCATCGG | *GGAAAAAAA*GGGTTTGTACATTGATTGCAGTCCTCCATA | ZnT | dsRNA temp |
| AAEL003964 | **TAATACGACTCACTATAGG**ACCTGTACTGGGCGTTCTTCATC | *GGAAAAAAA*CAACTCATCAATGGTCTGGAACG | ZnT | dsRNA temp |
| AAEL014902 | **TAATACGACTCACTATAGG**GGCTGCCCTTGTGATCTCCTTCCTAT | *GGAAAAAAA*GCAGTGGCCATACCGAGTTTAGAATC | ZnT | dsRNA temp |
| AAEL008693 | **TAATACGACTCACTATAGG**GCGATTGGCTTCACGCTTGTTTTTAT | *GGAAAAAAA*GCGTAGATCGTGGCGTTGAAGAC | ZnT | dsRNA temp |
| AAEL010511 | **TAATACGACTCACTATAGG**GCTATTTGCTCATCGGGGGCTACAC | *GGAAAAAAA*GGAAGAAGTTGATCACCATGTCGTTTATT | ZnT | dsRNA temp |
| AAEL010902 | **TAATACGACTCACTATAGG**GAGCGCTTTGCTTCAGCATAACGATA | *GGAAAAAAA*CAGCAGAATCAACGCCGACTCC | ZnT | dsRNA temp |
| AAEL011256 | **TAATACGACTCACTATAGG**GCGGTGCCATTCTGTCCATTGTG | *GGAAAAAAA*GCTGCGCGAACGTTGAGATTTT | ZnT | dsRNA temp |
| AAEL001626 | **TAATACGACTCACTATAGG**CTTCGACGCACCTGAAAGCAGTCAAAC | *GGAAAAAAA*CGGTTATGATCAGACTTAAAATAGA | ZIP | dsRNA temp |
| AAEL001968 | **TAATACGACTCACTATAGG**GAGAACAACAAAGTTTCCGACAAG | *GGAAAAAAA*GGAATGACGGACACGGTGGCAA | ZIP | dsRNA temp |
| AAEL013490 | **TAATACGACTCACTATAGG**AAACCCGCAGGATAACTCGTTGGC | *GGAAAAAAA*AGAATATCATCCGCCACAATGTAGAT | ZIP | dsRNA temp |
| AAEL014762 a | **TAATACGACTCACTATAGG**GATCCCGCCGAATCGAAGACACTCA | *GGAAAAAAA*TTCAGATAGCCGGCCACCTTTTTG | ZIP | dsRNA temp |
|  |  | **TAATACGACTCACTATAGGG**TTCAGATAGCCGGCCACCTTTTTG |  |  |
| AAEL014762 b | **TAATACGACTCACTATAGG**GCTGGTCTCGTTGGGGCGTTGGT | *GGAAAAAAA*CTATGGGCGGGGGCGAGGAT | ZIP | dsRNA temp |
|  |  | **TAATACGACTCACTATAGGG**CTATGGGCGGGGGCGAGGAT |  |  |
| AAEL005496 | **TAATACGACTCACTATAGG**GCGAGTTCGATGTGGACTAGGCAGTT | *GGAAAAAAA*GTCCAGTCCGTCCAGGGCCATTCCGATG | ZIP | dsRNA temp |
| AAEL014156 | **TAATACGACTCACTATAGG**GCAGGAGGCCGAACAGGACATTATTAG | *GGAAAAAAA*AAGCCCTTTGATCGATTTGTGCC | ZIP | dsRNA temp |
| AAEL010905 | **TAATACGACTCACTATAGG**GGAAACGAAGCACCACGGACACA | *GGAAAAAAA*TCGTACATCGCAATCAGAAGCAT | ZIP | dsRNA temp |
| AAEL007959 | **TAATACGACTCACTATAGG**GATTGTGATGCTGGTGGGGTCCTAC | *GGAAAAAAA*GGGGCCGACAGCGAGAAGAT | ZIP | dsRNA temp |
| AAEL008146 | **TAATACGACTCACTATAGG**GTGGCACAATGAAATATGGCAGGAA | *GGAAAAAAA*GGTATAAGCGCATGTGGAAGCAGAT | ZIP | dsRNA temp |
| AST15063.1 (EGFP) | **TAATACGACTCACTATAGG**GATGGTGAGCAAGGGCGAGGAGC | *GGAAAAAAA*ATCTTGAAGTTCACCTTGATGCCGTT | control | dsRNA temp |
|  |  | **TAATACGACTCACTATAGGG**ATCTTGAAGTTCACCTTGATGCCGTT |  |  |
| XM_001660119 | ACCGCCGTCTACGATGCCA | ATGGTGGTCTGCTGGTTCTT | rpS7 | qRT-PCR |
| AAEL000077 | GCTGGTCAATCTGGTCGGTA | TGTATCGCTGTGACCACCAT | ZnT | qRT-PCR |
| AAEL013490 | TCTTGGGAATCAACAGCCCa | GCGTCCATGTTCTAGCGAtG | ZIP | qRT-PCR |
| AAEL014762 | AAGGTGGCCGGCTATCTGAATCTG | CCACCAACGCCCCAACGAG | ZIP | qRT-PCR |

**Supplementary Table 1. Primers used in this study.** Undeline indicates restriction enzyme recognition sequence; bold letters indicate T7 promoter sequence; italic letters indicate Phi6 recognition sequence; FrLCH: Ferritin light chain; rpS7: ribosomal protein S7.

| **Species** | **Accession number** | **Gene ID (Flybase/VectorBase)** | **Common Name** | **Species** | **Accession number** | **Gene ID (Flybase/VectorBase)** | **Common Name** |
| --- | --- | --- | --- | --- | --- | --- | --- |
| *Ae. aegypti* | XP_001659748.1 | AAEL001626 |  | *Ae. aegypti* | XP_001647887.1 | **AAEL000077** | **AaeZnT7** |
| *Ae. aegypti* | XP_001660808.1 | AAEL001968 |  | *Ae. aegypti* | XP_001658531.1 | AAEL001239 |  |
| *Ae. aegypti* | XP_001650950.1 | AAEL005496 |  | *Ae. aegypti* | XP_001648127.1 | AAEL003964 |  |
| *Ae. aegypti* | XP_001658744.1 | AAEL007959 |  | *Ae. aegypti* | XP_001659422.1 | AAEL008693 |  |
| *Ae. aegypti* | XP_001658971.1 | AAEL008146 |  | *Ae. aegypti* | XP_001660896.1 | AAEL010511 |  |
| *Ae. aegypti* | XP_001661134.1 | AAEL010905 |  | *Ae. aegypti* | XP_001661141.1 | AAEL010902 |  |
| *Ae. aegypti* | XP_001663675.1 | **AAEL013490** | **AaeZIP11** | *Ae. aegypti* | XP_001661525.1 | AAEL011256 |  |
| *Ae. aegypti* | XP_001663959.1 | AAEL013756 |  | *Ae. aegypti* | XP_001649976.1 | AAEL014902 |  |
| *Ae. aegypti* | XP_001648141.1 | AAEL014156 |  | *An. gambiae* | XP_313758.4 | AGAP004461 |  |
| *Ae. aegypti* | XP_001649581.1 | **AAEL014762** | **AaeZIP13** | *An. gambiae* | XP_315949.4 | AGAP005918 |  |
| *An. gambiae* | XP_312308.5 | AGAP002624 |  | *An. gambiae* | XP_319731.4 | AGAP008982 |  |
| *An. gambiae* | XP_319524.2 | AGAP003300 |  | *An. gambiae* | XP_319754.4 | AGAP009005 |  |
| *An. gambiae* | XP_315414.4 | AGAP005405 |  | *An. gambiae* | XP_318514.3 | AGAP010794 |  |
| *An. gambiae* | XP_308163.4 | AGAP007713 |  | *An. gambiae* | XP_320480.3 | AGAP012046 |  |
| *An. gambiae* | XP_317427.4 | AGAP008034 |  | *H. sapiens* | NP_067017.2 | hZnT1 |  |
| *An. gambiae* | XP_319959.4 | AGAP009188 |  | *H. sapiens* | NP_001004434.1 | hZnT2 |  |
| *An. gambiae* | XP_309469.4 | AGAP011178 |  | *H. sapiens* | NP_003450.2 | hZnT3 |  |
| *An. gambiae* | XP_317935.4 | AGAP011388 |  | *H. sapiens* | AAB82561.1 | hZnT4 |  |
| *An. gambiae* | XP_307093.4 | AGAP012772 |  | *H. sapiens* | XP_005248626.1 | hZnT5 |  |
| *H. sapiens* | NP_055252.2 |  | hZIP1 | *H. sapiens* | NP_001180442.1 | hZnT6 |  |
| *H. sapiens* | AAF35832.1 |  | hZIP2 | *H. sapiens* | NP_598003.2 | hZnT7 |  |
| *H. sapiens* | NP_653165.2 |  | hZIP3 | *H. sapiens* | NP_776250.2 | hZnT8 |  |
| *H. sapiens* | NP_060237.2 |  | hZIP4 | *H. sapiens* | NP_006336.3 | hZnT9 |  |
| *H. sapiens* | NP_775867.2 |  | hZIP5 | *H. sapiens* | NP_061183.2 | hZnT10 |  |
| *H. sapiens* | NP_036451.3 |  | hZIP6 | *D. melanogaster* |  | CG6672 | dZnT86D |
| *H. sapiens* | NP_001275706.1 |  | hSLC39A7 | *D. melanogaster* |  | CG5130 | dZnT77C |
| *H. sapiens* | NP_001128620.1 |  | hZIP8 | *D. melanogaster* |  | CG3994 | dZnT35C |
| *H. sapiens* | NP_001239080.1 |  | hZIP9 | *D. melanogaster* |  | CG8632 | Znt49B |
| *H. sapiens* | XP_005246746.1 |  | hZIP10 | *D. melanogaster* |  | CG31860 | dZnT33D |
| *H. sapiens* | AAH65917.1 |  | hSLC39A12 | *D. melanogaster* |  | CG17723 | dZnT63 (dZnT1) |
| *H. sapiens* | NP_631916.2 |  | hZIP11 | *D. melanogaster* |  | CG11163 | dZnT44F |
| *H. sapiens* | NP_001121697.1 |  | hZIP13 |  |  |  |  |
| *H. sapiens* | NP_001121903.1 |  | hZIP14 |  |  |  |  |
| *D. melanogaster* |  | CG6898 | dZIP3 (dZIP89B) |  |  |  |  |
| *D. melanogaster* |  | CG6817 | foi |  |  |  |  |
| *D. melanogaster* |  | CG10449 | Catsup |  |  |  |  |
| *D. melanogaster* |  | CG13189 | dZIP48C |  |  |  |  |
| *D. melanogaster* |  | CG2177 | dZIP102B |  |  |  |  |
| *D. melanogaster* |  | CG7816 | dZIP13 (dZIP99C) |  |  |  |  |
| *D. melanogaster* |  | CG9430 | dZIP42C.2 |  |  |  |  |
| *D. melanogaster* |  | CG4334 | dZIP88E |  |  |  |  |
| *D. melanogaster* |  | CG10006 | dZIP71B |  |  |  |  |
| *D. melanogaster* |  | CG9428 | dZIP1 (dZIP42C.1) |  |  |  |  |

**Supplementary Table 2. Amino acid sequences used for phylogenetic analysis in Figure 4.** ZIPs on the left, and ZnTs on the right. Yellow-Highlighted information was used for tip labels for the phylogenetic trees.
